# Functional Significance of Allelic Variation at *methuselah*, an Aging Gene in *Drosophila*

**DOI:** 10.1371/journal.pone.0001987

**Published:** 2008-04-16

**Authors:** Annalise B. Paaby, Paul S. Schmidt

**Affiliations:** Department of Biology, University of Pennsylvania, Philadelphia, Pennsylvania, United States of America; Indiana University, United States of America

## Abstract

**Background:**

Longevity and age-specific patterns of mortality are complex traits that vary within and among taxa. Multiple candidate genes for aging have been identified in model systems by extended longevity mutant phenotypes, including the G-protein coupled receptor *methuselah* (*mth*) in *D. melanogaster*. These genes offer important insights into the mechanisms of lifespan determination and have been major targets of interest in the biology of aging. However, it is largely unknown whether these genes contribute to genetic variance for lifespan in natural populations, and consequently contribute to lifespan evolution.

**Methodology/Principle Findings:**

For a gene to contribute to genetic variance for a particular trait, it must meet two criteria: natural allelic variation and functional differences among variants. Previous work showed that *mth* varies significantly among wild populations; here we assess the functional significance of wild-derived *mth* alleles on lifespan, fecundity and stress resistance using a quantitative complementation scheme. Our results demonstrate that *mth* alleles segregating in nature have a functional effect on all three traits.

**Conclusions/Significance:**

These results suggest that allelic variation at *mth* contributes to observed differences in lifespan and correlated phenotypes in natural populations, and that evaluation of genetic diversity at candidate genes for aging can be a fruitful approach to identifying loci contributing to lifespan evolution.

## Introduction

Lifespan and age specific mortality rates are primary life history components and vary significantly among natural populations [1]. QTL and artificial selection experiments have demonstrated a highly complex genetic architecture, with many genetic correlations among lifespan and associated life history traits [e.g. 2], [3]. Nonetheless, single gene manipulations have identified candidate genes for aging by extended longevity phenotypes, and in the model system *Drosophila melanogaster* such genes include the *Insulin-like Receptor* [4], *chico* [5], *dFOXO* [6], *Indy* [7] and *methuselah* [8].

Williams' [9] theory of antagonistic pleiotropy describes how pleiotropic alleles that increase fitness early in life may experience positive selection even though they incur a fitness cost later in life. Identified aging genes have consistently shown costs to lifespan extension, particularly in reproduction. Although there is some evidence that lifespan and reproductive success can be decoupled [10], multiple analyses have revealed previously undetected tradeoffs under specific conditions [e.g. 11], [12], [13]. In addition to demonstrating negative effects on reproduction, longevity mutations are positively correlated with stress resistance [14]. Such correlations may explain aspects of lifespan evolution, and why loss-of-function mutants can result in lifespan extension. However, it remains unclear whether identified aging genes are major contributing factors to the genetic variance for longevity that is routinely observed in populations [e.g. 15]. The role of aging genes, such as the components of the insulin (IGF-1/IIS) pathway, are highly conserved in metazoans [16]. Such genes may experience strong selection constraints and consequently exhibit little variation at the nucleotide level. Furthermore, a gene may be polymorphic, but the variation may be functionally neutral. In order to comprehensively examine the contribution of candidate genes for aging to the genetic variance for longevity and correlated traits, the functional significance of allelic variation must be assessed.

The G-protein coupled receptor *methuselah* (*mth*) is a promising candidate for such analyses. This gene, discovered by its extended longevity mutant phenotype, was the first candidate gene for aging identified in *D. melanogaster*. Individuals homozygous for a P-element disruption at *mth* lived an average of 35% longer than the parental strain and showed significant resistance to oxidative stress, starvation and heat stress [8]. Mutants also show a tradeoff in lifetime reproductive success under heat stress conditions [13]. The gene encodes a G-protein coupled receptor, showing seven hydrophobic regions suggestive of transmembrane domains [8] and an ectodomain containing a ligand binding site [17]. Disruption of *mth* ligand activity also promotes lifespan: mutation at *stunted*, a gene that produces two *mth* peptide ligands, and constitutive expression of antagonist peptide ligands both produce extensions in longevity [18], [19]. Mutants show a reduction in excitatory neurosecretion, and *mth* appears to modulate synaptic strength in neurons by regulating vesicle trafficking [20]; this may be important in sensorimotor ability, as *mth* mutants also show enhanced visuomotor synchronization and phototaxis [21]. Despite these implications for neuroendocrine signaling, the mechanism of lifespan regulation by *mth* is not well understood. The normal reduction in germline stem cell division is not exhibited in aging *mth* mutants [22], revealing a potentially different process by which *mth* affects physiology. It may be that *mth* is pleiotropic both in the traits it determines and the mechanisms by which determination happens.

In addition to results demonstrating its function as a pleiotropic aging gene, data from the wild suggest that *mth* may be an important component in lifespan evolution. In a comparison of expression levels for *D. melanogaster*, *D. simulans* and their F1 hybrids for 31 genes, *mth* was shown to have one of the strongest patterns for compensatory *cis-trans* regulatory evolution [23], suggesting that *mth* has experienced strong selection on expression level since *D. melanogaster* and *D. simulans* shared a common ancestor approximately two million years ago. Over this same timescale, *mth* also shows a very high rate of protein evolution [24]. Furthermore, wild populations of *D. melanogaster* show a cline in the frequency of the most common *mth* haplotype along the latitudinal gradient of the U.S. east coast [24], a pattern that decays with decreasing linkage disequilibrium both 5′ and 3′ of the *mth* locus [25]. This pattern covaries with clines in longevity, fecundity, stress resistance and other life history traits in these populations [26]. Genetic variance for and genetic correlations among these traits underlie predictable life history variation that reflects distinct selection pressures that vary spatially and temporally [27], [28], [29]. Together, these results imply that *mth* has experienced directional selection pressures over short and long timescales, and that *mth* may be an important target in the selection regime driving the observed patterns of life history variation in natural populations. However, it is unknown whether the observed allelic variation at *mth* is of functional significance, and contributes to the genetic variance for lifespan in natural populations.

Here we present results from a modified quantitative complementation scheme [30], in which we tested whether a set of wild-derived *mth* alleles show differences in lifespan, fecundity and resistance to oxidative stress.

## Materials and Methods

### Quantitative complementation scheme

We used a modified quantitative complementation scheme [30] to test the contribution to phenotype of eight wild *mth* alleles still embedded in their natural genomes. Each wild line was crossed to one of two mutant *mth* alleles, and the phenotype of this genetic construct was compared to the phenotype of the same wild line over a functional, wild-type *mth* allele. The magnitude of difference among line pairs was then used to test for differences among lines. The use of two mutant *mth* alleles generated two independent complementation tests for each line in each of three trait assays.

Each of the eight wild-derived lines was crossed to the three lab-derived *mth* alleles, *mth^1^*, *mth^R3^* and *mth^Δ6^*. All parental lines were maintained at low density on standard cornmeal-molasses medium in bottle cultures to limit confounding environmental variation. In the crosses with *mth^Δ6^*, which is homozygous lethal, F1 pupae not exhibiting the balancer phenotype were selected prior to eclosion. The F1 progeny used for the assays were of the following genotypes: *+_i_*/*mth^Δ6^*, *+_i_*/*mth^1^* and *+_i_*/*mth^R3^*, where *+_i_* is one of the eight wild-derived lines. These three genotypic categories allowed us to compare the phenotype of each *+_i_* line over a *mth* mutant (either *mth^Δ6^* or *mth^1^*) to the phenotype of that same line over a functional copy of *mth* (*mth^R3^*). In the statistical analyses, there were two criteria for establishing differences among the wild *mth* alleles. First, significant interaction terms between the wild lines and the lab-derived *mth* alleles were used to indicate a failure to complement, or significant functional variation in the wild lines. Second, an F-statistic was used to ensure that the variance in the wild-type background was not greater than variance in the mutant backgrounds; greater variance in the wild-type background would suggest that epistatic interaction between the wild lines and the wild-type *mth^R3^* allele, rather than allelic variation, produced the failure to complement [31].

### Flies

Approximately 40 isofemale lines were established from wild populations in Maine, New Jersey, Pennsylvania and Florida [described in 29]. Flies were made isogenic at the third chromosome using balancers and a ∼1 kb region of *mth* was sequenced to characterize each line by haplotype as determined by SNP identity [24], [25]. The two remaining chromosomes were not background-replaced or otherwise made isogenic within or between lines. We confirmed that the frequency of the common *mth* haplotype in our three geographic regions was consistent with the cline in frequency demonstrated previously [24]. Eight lines (referenced here as *BF51*, *BF54*, *RR5*, *S108*, *S97*, *SL5135*, *T28* and *T50*) were selected for the functional tests, based on this haplotype identity as well as polymorphism at the remaining sites within the sequenced ∼1 kb region. All the polymorphisms we observed in this region were consistent with the polymorphisms reported earlier [see 24], with the exception of four additional singleton SNPs. Consequently the eight wild lines selected were unique according to their sequence identity within this region.

The lab-derived *mth* alleles include three *mth* genotypes in a standardized background [8], [20]: a P-element insertion hypomorph (*mth^1^*), a *mth* null (*mth^Δ6^*), and a wild-type *mth* allele (*mth^R3^*). The *mth^1^* hypomorph was generated by insertion of the standard P{lacW} element in the *w1118* background [8]. Both the null and the wild-type alleles were created by excision of the P-element in the original *mth^1^* line. The *mth^Δ6^* null allele resulted from an imprecise excision that removed the C-terminal end of exon 3, the N-terminal end of exon 4, and the intron between exons 3 and 4 [20]. The *mth^R3^* wild-type revertant allele resulted from a precise excision of the P-element, which restored wild-type function [8], [20]. The *mth^Δ6^* allele is homozygous lethal, and was maintained over a TM6 balancer.

### Functional assays

For the lifespan and fecundity assays, flies were collected, freshly eclosed, over three days. Flies experienced a short, single exposure to CO_2_ during virgin/male sorting. For each of the 24 genotypic combinations, demography cages were initiated by putting 40 males and 40 females of a single 24-hour cohort into perforated 6-oz polypropylene bottles. Each combination of wild line by *mth* allele was replicated three times. Cornmeal-molasses food plates were changed every day for the first 16 days and every other day afterwards. Eggs laid and dead flies were scored at every plate change. Cages were kept at room temperature. For the oxidative stress assay, flies were collected, freshly eclosed, over an 8-hour window, using a short, single exposure to CO_2_. For each genotypic combination, five males and five females were put in vials with standard cornmeal-molasses media and aged for four days. Each vial was replicated four times. Flies were then transferred into media-free vials with cotton saturated with 5 mL of 30 mM methyl viologen (paraquat) in 5% sucrose solution. After 19 hours of continuous exposure, patterns of mortality were determined for all replicates.

### Statistics

Our analyses tested the effects of line, allele, sex and the interactions among these effects. Here, as elsewhere, “line” refers to the eight wild-derived variants and “allele” refers to the three lab-derived *mth* alleles. Lifespan data for each sex were also analyzed separately and there was no sex term in the fecundity analysis. For the lifespan data, a proportional hazards model was used to test for main and interaction effects and to estimate risk ratios. Total fecundity was analyzed with ANOVA, treating the line and line by allele interaction as random effects. The oxidative stress data were analyzed with nominal logistic regression, modeling the log odds (mortality/survivorship). Because the wild lines varied at multiple loci, epistasis between these other genes derived from natural populations and the lab-derived wild-type *mth* allele may have contributed confounding effects. To test whether epistatic interactions between genes on the wild-derived chromosomes and the *mth^R3^* background were responsible for the failures to complement, we used an F-statistic to examine the equivalency of variances in both the *mth* wild-type and mutant backgrounds [31]. Variance components were computed as sums of squares for each genotype (*mth^R3^*, *mth^Δ6^* and *mth^1^*) for each of the functional assays. For each comparison, F = Var(*mth^R3^*/*+_i_*)/Var(*mth^Δ6^/+_i_* or *mth^1^/+_i_*). F values lower than the critical value for a type I error of 0.05 allowed us to accept the null hypothesis, that variance in the *mth^R3^* background was not significantly greater than variance in the mutant background and consequently not responsible for observed differences among lines. All statistical procedures were performed using JMPv5 (SAS Institute, Cary, NC).

## Results

The wild *mth* lines exhibited functional differences in lifespan, fecundity and resistance to oxidative stress. These differences were demonstrated in both of our independent complementation schemes, which each used a different mutant *mth* allele to measure the wild *mth* variants' contribution to phenotype. Differences in function among wild *mth* lines were seen in the magnitudes of difference between lines over the mutant *mth* alleles (*mth^Δ6^*, the deletion, or *mth^1^*, the P-element hypomorph) and these same lines over a functional *mth* allele (*mth^R3^*, the wild-type revertant), or by a significant line by allele interaction term in the statistical analysis. Qualitative comparisons between the two independent tests showed similarities in which lines fail to complement, the directionality of complementation failure, and the significance of effect and interaction terms. For each functional assay, the variance in the *mth^R3^* wild-type background was not significantly greater than the variance in either the *mth^Δ6^* or *mth^1^* mutant backgrounds, which supports allelism over epistasis as the genetic mechanism generating the differences in phenotype among lines.

### Lifespan

The eight wild *mth* lines showed phenotypic variation in lifespan in both the *mth^Δ6^* and the *mth^1^* complementation tests (Table 1). Additionally, females and males exhibited sex-specific differences in *mth* allelic contribution to lifespan. As expected, the three lab-derived *mth* alleles, *mth^Δ6^*, *mth^1^* and *mth^R3^* showed variation in longevity as well. Since reduced *mth* expression is associated with lifespan extension [8], we predicted that the *mth^Δ6^* and *mth^1^* mutants would exhibit increased longevity relative to the *mth^R3^* wild-type allele. However, this was not observed: the *mth^1^* allele exhibited longest lifespan, but the *mth^Δ6^* allele exihibited shortest lifespan (Table S1) . The significant line by allele interaction terms, which indicate that wild *mth* variants produce functional differences in longevity, were consistent across the two complementation tests (Table 1). This suggests that the observed results were not due to idiosyncratic effects of the various *mth* mutants or interactions with the wild *mth* lines. Furthermore, the phenotypic differences among wild *mth* lines did not result from epistatic effects between the functional *mth^R3^* allele and the wild-derived lines, because the variation in the wild-type background was not significantly greater than the variation in the mutant backgrounds (Table 1).

**Table 1:** Statistical results for the lifespan assay.

*Proportional hazards model effect likelihood ratio tests*							
test using *mth^Δ6^* and *mth^R3^*				test using *mth^1^* and *mth^R3^*			
Source	DF	χ^2^	p	Source	DF	χ^2^	p
line	1	213.966	<0.0001	line	1	143.172	<0.0001
allele	7	73.708	<0.0001	allele	7	69.211	<0.0001
sex	1	6.609	0.0101	sex	1	10.956	0.0009
line×allele	7	36.517	<0.0001	line×allele	7	27.352	0.0003
line×sex	1	157.598	<0.0001	line×sex	1	211.332	<0.0001
allele×sex	7	8.604	0.0034	allele×sex	7	7.601	0.0058
line×allele×sex	7	35.610	<0.0001	line×allele×sex	7	34.726	<0.0001

*F-statistic testing variances for age at death among alleles*							
test using *mth^Δ6^* and *mth^R3^*				test using *mth^1^* and *mth^R3^*			
Ratio	DF	F	critical F	Ratio	DF	F	critical F
σ^2^ *_mthR3/+i_*/σ^2^ *_mthΔ6/+i_*	7, 7	1.2511 (f)	3.787	σ^2^ *_mthR3/+i_*/σ^2^ *_mth1/+i_*	7, 7	1.5810 (f)	3.787
		0.1375 (m)				0.1887 (m)	

**Figure 1:**
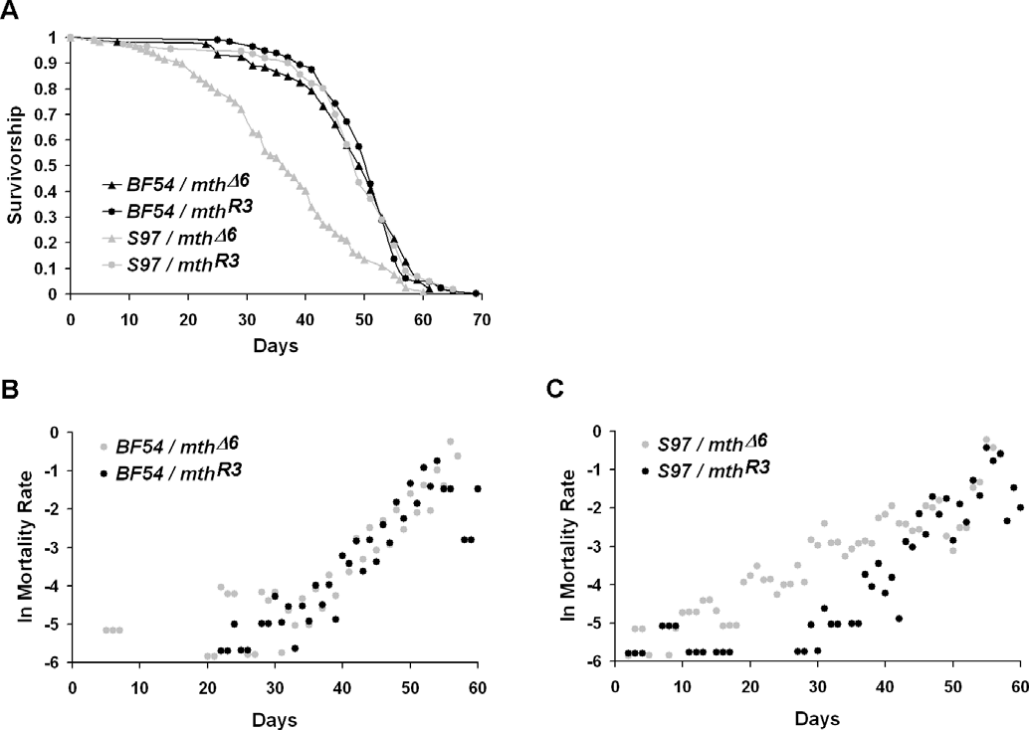
A sample comparison of paired survivorship curves and paired mortality rates for two wild lines. Males from lines *BF54* and *S97* show differences in survivorship, as *BF54* over *mth^Δ6^* survived the same as *BF54* over *mth^R3^,* while *S97* over *mth^Δ6^* survived more poorly than *S97* over *mth^R3^* (A). These lines also exhibit differences in mortality rate: *BF54* shows the same mortality rate when paired with *mth^Δ6^* as when paired with *mth^R3^* (B), while *S97* shows a higher mortality rate when paired with *mth^Δ6^* than when paired with *mth^R3^* (C).

**Figure 2:**
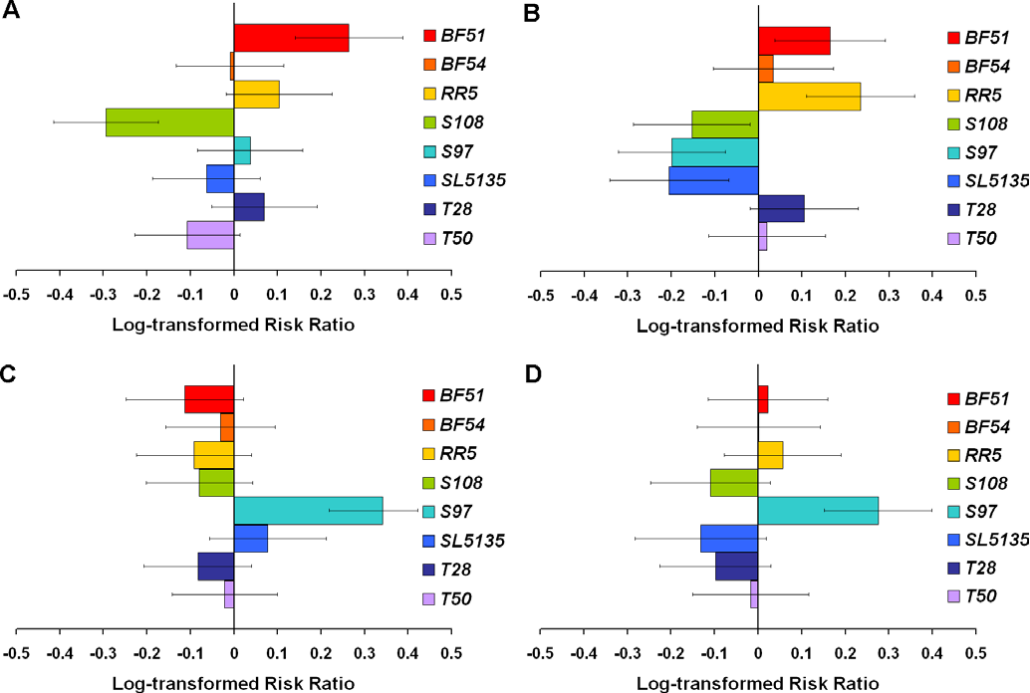
Risk ratios for each line over one of the *mth* mutants relative to that line over *mth^R3^*. The comparisons using *mth^Δ6^* are on the left (A and C); the comparisons using *mth^1^* are on the right (B and D). Females are above (A and B), males below (C and D). Positive ratios indicate greater risk of death, so the positive ratios for *BF51* females (A and B) mean that this line showed lower expected lifespan over the *mth* mutants than over the *mth* wild-type allele. The heterogeneity in the magnitude and direction of relative risk represents the functional diversity in lifespan among the wild *mth* lines. Error bars show 95% confidence. Error bars which do not cross the y-axis signify a failure to complement in that line.

Variation in longevity among the wild lines can be seen in a sample comparison of paired survivorship curves and mortality rates (Figure 1). For example, the difference in survivorship between line *S97* over *mth^Δ6^* and over *mth^R3^* is greater than the difference in survivorship between line *BF54* over *mth^Δ6^* and over *mth^R3^* (Figure 1A). In this case, the single copy of *mth* in line *S97* failed to complement, shown by the difference in longevity between this line over the *mth^Δ6^* deletion and the *mth^R3^* wild-type allele; line *BF54* complemented, with no difference in longevity when paired with *mth^Δ6^* than when paired with *mth^R3^*. The *S97* and *BF54* lines also aged differently. Line *S97* showed no difference in mortality rate when over the *mth^Δ6^* allele than when over *mth^R3^* (Figure 1B), but line *BF54* showed a higher mortality rate when paired with *mth^Δ6^* than when paired with *mth^R3^* (Figure 1C). Risk ratios generated from the proportional hazards analysis illustrate the heterogeneity in longevity among all the natural *mth* lines (Figure 2). The risk ratios show the risk of death for each wild line over a *mth* mutant (*mth^Δ6^*, Figure 2A and 2C, or *mth^1^*, Figure 2B and 2D) relative to that same line over the functional *mth* allele (*mth^R3^*). Some lines survived longer in the wild-type background, while others survived longer in the mutant background; this is shown by positive ratios for some lines and negative ratios for others (e.g. lines *BF51* and *S108*, Figure 2A and 2B). Females (Figure 2A and 2B) and males (Figure 2C and 2D) are shown separately, as females and males were affected differently by allelic contribution to longevity (Table 1).

### Fecundity

**Figure 3:**
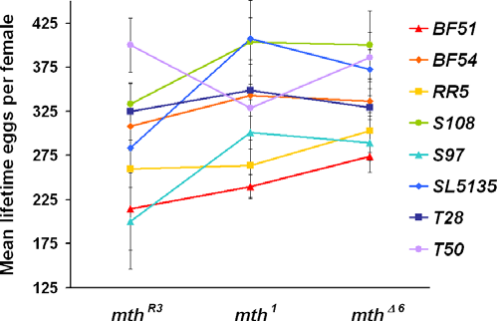
Average lifetime fecundity per line for all genotypic combinations. Non-parallel slopes between lines in the three lab-derived *mth* backgrounds indicate the functional variation in lifetime fecundity among the wild lines. The general increase in eggs laid across the x-axis represents a correlation of increasing fecundity with decreasing background *mth* expression; all lines but *T50* showed lowest fecundity when paired with the wild-type *mth^R3^* allele. Error bars show 95% confidence.

The wild *mth* lines showed functional differences in total lifetime fecundity for both complementation tests (Table 2, Figure 3), and an analysis of fecundity per female per day yielded quantitatively identical results (data not shown). The fecundity assays also demonstrated, predictably, an effect by *mth* allele; unpredictably, the *mth^Δ6^* allele showed higher fecundity than *mth^R3^*, and the comparison between *mth^1^* and *mth^R3^* was nonsignificant (Table 2, Table S1). Comparison of variation among *mth* allele backgrounds supports allelism, as variation in the wild-type background was not greater than variation in the mutant backgrounds (Table 2).

**Table 2:** Statistical results for the fecundity assay.

*ANOVA of lifetime fecundity*									
test using *mth^Δ6^* and *mth^R3^*					test using *mth^1^* and *mth^R3^*				
Source	DF	SS	F	p	Source	DF	SS	F	p
line	7	1.935e8	8.100	0.0066	line	7	1.874e8	3.586	0.0569
allele	1	4.051e7	11.874	0.0108	allele	1	2.601e7	3.515	0.1028
line×allele	7	2.388e7	2.900	0.0182	line×allele	7	5.227e7	6.197	0.0002

*F-statistic testing variances for total eggs laid among alleles*							
test using *mth^Δ6^* and *mth^R3^*				test using *mth^1^* and *mth^R3^*			
Ratio	DF	F	critical F	Ratio	DF	F	critical F
σ^2^ *_mthR3/+i_*/σ^2^ *_mthΔ6/+i_*	7, 7	2.0146	3.787	σ^2^ *_mthR3/+i_*/σ^2^ *_mth1/+i_*	7, 7	1.4202	3.787

### Stress resistance

**Figure 4:**
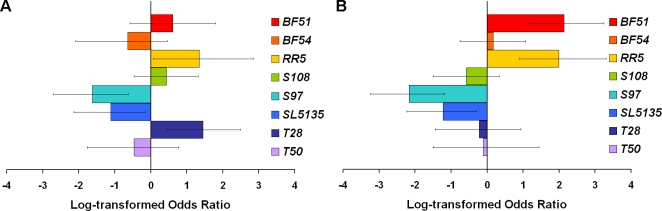
Odds ratios for each line over one of the *mth* mutants (*mth^Δ6^* or *mth^1^*) relative to the line over *mth^R3^*. The comparison using *mth^Δ6^* is on the left (A); the comparison using *mth^1^* is on the right (B). Positive ratios indicate greater risk of death. The heterogeneity in the magnitude and direction of relative odds represents the functional diversity in stress resistance among the wild *mth* lines. Error bars show 95% confidence. Error bars which do not cross the y-axis signify a failure to complement in that line.

The wild *mth* lines also demonstrated differences in response to oxidative stress in both complementation tests (Table 3). Odds ratios illustrate the variation in resistance to oxidative stress among lines (Figure 4). Analogous to the risk ratios in the lifespan analyses, these ratios show the odds of death for each line over one of the *mth* mutants (*mth^Δ6^*, Figure 4A; *mth^1^*, Figure 4B) relative to that same line over the functional *mth* allele (*mth^R3^*). Data were pooled across sexes, as we observed no effect of sex on allele function in this assay (Table 3). Like the lifespan risk ratios, the oxidative stress odds ratios demonstrate extensive heterogeneity among the wild lines: some lines showed better performance in the wild-type background (a positive odds ratio), whereas some lines showed better performance in the mutant background (a negative odds ratio). For example, line *RR5* was more resistant to oxidative stress when paired with the functional *mth^R3^* allele than with either of the mutant alleles, *mth^Δ6^* or *mth^1^*; however, lines *S97* and *SL5135* were both more resistant to oxidative stress when paired with *mth^Δ6^* and *mth^1^* than with *mth^R3^* (Figure 4A and 4B). Comparison across the lifespan and oxidative stress assays suggests a positive correlation between these traits among females. Four lines show significant differences in both assays: *BF51* and *RR5* show increased stress resistance and longer lifespan in the wild-type background; *S97* and *SL5135* show increased stress resistance and longer lifespan in the mutant backgrounds; no line showed increased stress resistance but shorter lifespan (or vice versa) in the same background. Like the lifespan and fecundity assays, the differences among lines in resistance to oxidative stress suggests allelism, as variation in the wild-type background was not greater than variation in the mutant backgrounds (Table 3). As predicted, the *mth^Δ6^* allele did show greater resistance to oxidative stress than the wild-type *mth^R3^* allele; the comparison using the *mth^1^* allele was nonsignificant.

**Table 3:** Statistical results for the oxidative stress resistance assay.

*Nominal logistic model effect Wald tests*							
test using *mth^Δ6^* and *mth^R3^*				test using *mth^1^* and *mth^R3^*			
Source	DF	χ^2^	p	Source	DF	χ^2^	p
line	1	95.899	<0.0001	line	1	87.944	<0.0001
allele	7	11.812	0.0006	allele	7	0.828	0.3629
sex	1	0.015	0.9039	sex	1	0.899	0.3429
line×allele	7	26.799	0.0004	line×allele	7	46.216	<0.0001
line×sex	1	18.390	0.0103	line×sex	1	8.980	0.2541
allele×sex	7	1.189	0.2755	allele×sex	7	3.761	0.0525
line×allele×sex	7	7.695	0.3603	line×allele×sex	7	10.096	0.1832

*F-statistic testing variances for flies dead at hour 19 among alleles*							
test using *mth^Δ6^* and *mth^R3^*				test using *mth^1^* and *mth^R3^*			
Ratio	DF	F	critical F	Ratio	DF	F	critical F
σ^2^ *_mthR3/+i_*/σ^2^ *_mthΔ6/+i_*	7, 7	3.711	3.787	σ^2^ *_mthR3/+i_*/σ^2^ *_mth1/+i_*	7, 7	2.148	3.787

## Discussion

Our results demonstrate that natural variants of *mth*, a gene for aging in *Drosophila*, contribute to significant functional variation in lifespan, fecundity and resistance to oxidative damage. In the lifespan analyses, the significant line by allele by sex interaction term also demonstrates that variation at *mth* affects males and females differently. This observation is consistent with the sex-specific effects of identified QTLs for lifespan [32] and complementation data for other candidate genes for aging [33]. Furthermore, the observed data do not result from idiosyncrasies associated with a particular *mth* mutant allele, as the patterns were qualitatively identical between the two complementation tests. Nor does the failure to complement appear to be determined by epistatic interaction between the functional lab-derived wild-type allele and the wild-derived lines, as the F ratio tests were nonsignificant for all comparisons. Other studies have demonstrated the significance of genetic background and epistatic interactions on patterns of longevity [34], [33], [35]. In this study, the standardization of the genetic background reduces the likelihood of identifying interesting epistatic interactions among genes affecting longevity, but may increase the power to detect small differences in function among naturally-occurring wild-type variants.

While our data support the hypothesis that allelic variation at *mth* contributes to the genetic variance for longevity in natural populations, the nature of the complementation scheme precludes identifying which specific *mth* variants are associated with relative lifespan extension. This results from the fact that the assayed *mth* variants are embedded in their own genetic backgrounds, and the tests of functional significance evaluate the heterogeneity among line by allele genotypic crosses. As such, these analyses do not test the hypothesis that wild *mth* alleles show a trend in *mth* function by geography, but that diverse *mth* alleles (derived from geographically diverse populations) are functionally distinct. However, other trends do emerge from the data. Interestingly, the *mth* mutants did not show consistently longer lifespan or lower fecundity than the wild-type genotypes, as the initial characterization of *mth* as a longevity gene may have predicted. The original functional assays for *mth* showed a lifespan extension in flies homozygous for *mth^1^* and an increased resistance to oxidative stress in flies heterozygous for *mth^1^* and *mth^Δ6^* [8]; a tradeoff with fecundity was later demonstrated at higher temperatures [13]. Consequently our results are consistent with the earlier observation that lifespan extension is not achieved by the *mth^Δ6^* mutation, although it challenges the hypothesis that there is a straightforward relationship between *mth* expression and lifespan. For example, it is possible that the *mth^Δ6^* allele showed shorter lifespan than the wild-type *mth^R3^* allele (Table S1) because too great a reduction in *mth* expression compromises overall fitness due to the pleiotropic nature of the gene. However, it is also possible that the *mth^Δ6^* mutation affects more than just the *mth* locus; a deleterious effect by another gene would likely compromise longevity. The fact that the *mth^Δ6^* allele showed predictably higher oxidative stress tolerance (Table S1) but unpredictably shorter lifespan also suggests that the highly quantitative determination of lifespan can complicate attempts to interpret mechanisms of genetic control.

Our results are consistent with data showing that lifespan extension by *mth* is limited when males and females are permitted to mate [36], since all our assays were conducted with mixed-sex replicates. Our results also support the conclusion that lifespan extension and life history phenotypes associated with longevity genes are highly dependent upon genetic and environmental context. The overexpression of *superoxide dismutase* (*SOD*) was originally shown to increase longevity and stress resistance [37], but these phenotypes were later demonstrated to be genotype- and sex-specific when *SOD* was overexpressed in naturally long-lived genetic backgrounds [34]. The lifespan extension of *mth* mutants is also dependent upon genetic background and sex, and the reduced fecundity of *mth* mutants was only revealed under exposure to environmental stress [13]. These results are consistent with our data, which show a striking effect by sex in the lifespan assay.

The mechanisms by which variation at *mth* affects performance are unknown, but we hypothesize that differences in gene expression may drive the observed functional variation among *mth* alleles. Haplotypes at the *mth* locus demonstrate a significant latitudinal cline in frequency that mirrors differences among populations in expected lifespan, but none of the individual polymorphic sites at *mth* exhibit a clinal pattern [24]. This cline decays in both directions away from the *mth* locus, indicating that the actual site(s) under selection reside not in the coding region, but possibly in promoter or regulatory regions [25]. Expanded sequencing of previously characterized *mth* variants has revealed high polymorphism in the 5′ and 3′ *mth* UTR, and the potential functional impact of these polymorphisms on expression level is currently being explored. Alternatively, functional differences among wild *mth* variants may be caused by distinct properties of the protein if the mechanism is more complex than a single amino acid polymorphism. We are currently evaluating this possibility by examining patterns of linkage disequilibrium and geographical distribution in our expanded *mth* sequence dataset.

In addition to identifying the pathways and genes that regulate aging, determining the underlying genetic basis for differences in lifespan among individuals within populations is critical for understanding how a quantitative trait evolves. The significant and predictable variation in life histories among *Drosophila* populations provides an opportunity to dissect these differences in nature while affording all the advantages of a model organism. Longevity is highly variable within *D. melanogaster* [1], and wild populations show genetically correlated differences in lifespan and other life history traits by environment [38], [29]. This standing genetic variance has enabled mapping of lifespan QTL [3], [39] and led to precise identification of additional candidate genes for aging [33], [40]. Several studies have found functional significance of allelic variation at lifespan QTL [31] and candidate genes [40], [15], confirming that these loci may contribute to the observed variation in longevity phenotypes. However, in order to comprehensively describe the contribution of any one gene to lifespan evolution, knowledge about functional allelic variation must be integrated with patterns environmental heterogeneity and possible selection pressures in the wild.

By examining the functional significance of allelic variation at a single locus within the context of environmental heterogeneity, our study provides a complementary approach to the evaluation of a quantitative trait by QTL analysis. The significance of functional variation at *mth* is reinforced by the adaptive pattern this allelic variation exhibits among natural populations, and suggests that *mth* may be an important component in lifespan evolution. By testing natural variation at this gene, we have also demonstrated the utility of forward genetics in identifying loci that contribute to the evolution of a complex quantitative trait. Ultimately, differences in expected lifespan among individual genotypes will be resolved by the joint processes of gene identification, characterization of molecular mechanisms, and associations between specific variants and phenotypes.

## Supporting Information

Table S1 Phenotype means for the three lab-derived *mth* alleles.
(0.03 MB DOC)
