# Supplementary material for: Functional Significance of Allelic Variation at methuselah, an Aging Gene in Drosophila
Source: PLoS One. 2008 Apr 16;3(4):e1987. doi: 10.1371/journal.pone.0001987 (PMC2288678; doi:10.1371/journal.pone.0001987)
Supplement: Table S1 — Phenotype means for the three lab-derived mth alleles. (0.03 MB DOC) [file pone.0001987.s001.doc]

TABLE S1. Phenotype means for the three lab-derived *mth* alleles.

| *Age death (days)* | | | | |
| --- | --- | --- | --- | --- |
|  | Mean | St dev | St err mean | N |
| *mth^R3^* females | 46.89 | 13.73 | 0.46 | 901 |
| *mth^R3^* males | 48.22 | 10.98 | 0.39 | 810 |
| *mth^1^* females | 50.25 | 14.52 | 0.53 | 751 |
| *mth^1^* males | 49.09 | 11.67 | 0.44 | 714 |
| *mth^Δ6^* females | 43.32 | 12.80 | 0.42 | 928 |
| *mth^Δ6^* males | 44.49 | 12.95 | 0.43 | 889 |
|  | | | | |
| *Lifetime eggs laid per female* | | | | |
|  | Mean | St dev | St err mean | N |
| *mth^R3^* | 290.5 | 67.6 | 13.8 | 24 |
| *mth^1^* | 321.4 | 60.3 | 13.5 | 20 |
| *mth^Δ6^* | 336.5 | 48.6 | 9.9 | 24 |
|  | | | | |
| *Proportion of flies dead at hour 19 after exposure to paraquat* | | | | |
|  | Mean | St dev | St err mean | N |
| *mth^R3^* | 0.4844 | 0.3139 | 0.1110 | 8 |
| *mth^1^* | 0.4344 | 0.2142 | 0.0757 | 8 |
| *mth^Δ6^* | 0.3313 | 0.1630 | 0.0567 | 8 |

The sample sizes (N) for age at death represent individual flies. N would sum to 960 for each *mth* allele (40 individuals of each sex per bottle x 8 wild lines x 3 replicate bottles per allele/line genotype), except that flies were occasionally lost as escapees and four *mth^1^* bottles (a total of 160 flies of each sex) were excluded because of massive escape events. The sample sizes for eggs laid represent the total number of bottles for each allele (8 wild lines x 3 replicates); each bottle held 40 females and the same four *mth^1^* bottles were excluded. The sample sizes for flies dead after paraquat exposure represent the eight wild lines over which each *mth* allele was tested; each allele/line genotype was replicated five times, with five males and five female per replicate vial.
